# The tail of cryptochromes: an intrinsically disordered cog within the mammalian circadian clock

**DOI:** 10.1186/s12964-020-00665-z

**Published:** 2020-11-16

**Authors:** Gian Carlo G. Parico, Carrie L. Partch

**Affiliations:** 1 Department of Chemistry and Biochemistry, UC Santa Cruz, Santa Cruz, USA; 2 Center for Circadian Biology, UC San Diego, La Jolla, USA

**Keywords:** Cryptochrome, C-terminal tail, Circadian rhythms, Autoinhibition, Intrinsically disordered region, Intrinsically disordered protein

## Abstract

Cryptochrome (CRY) proteins play an essential role in regulating mammalian circadian rhythms. CRY is composed of a structured N-terminal domain known as the photolyase homology region (PHR), which is tethered to an intrinsically disordered C-terminal tail. The PHR domain is a critical hub for binding other circadian clock components such as CLOCK, BMAL1, PERIOD, or the ubiquitin ligases FBXL3 and FBXL21. While the isolated PHR domain is necessary and sufficient to generate circadian rhythms, removing or modifying the cryptochrome tails modulates the amplitude and/or periodicity of circadian rhythms, suggesting that they play important regulatory roles in the molecular circadian clock. In this commentary, we will discuss how recent studies of these intrinsically disordered tails are helping to establish a general and evolutionarily conserved model for CRY function, where the function of PHR domains is modulated by reversible interactions with their intrinsically disordered tails.

Video abstract

**Supplementary information:**

**Supplementary information** accompanies this paper at 10.1186/s12964-020-00665-z.

## Background

**Fig. 1:**
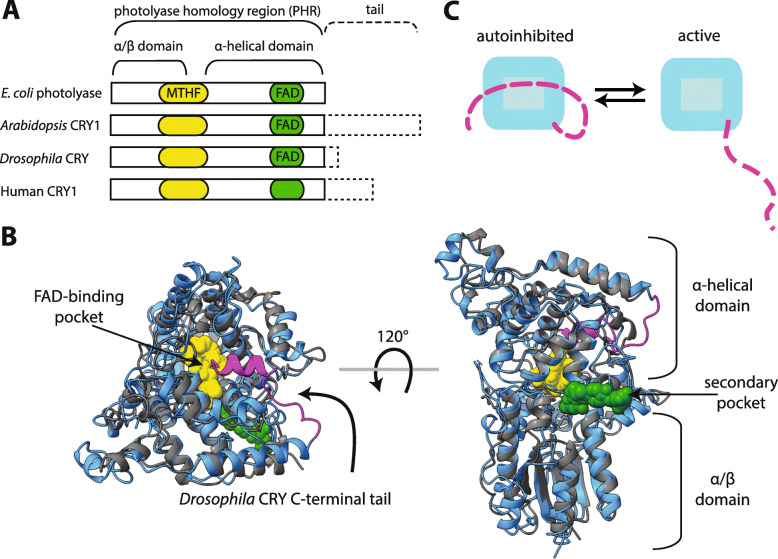
The photolyase/cryptochrome PHR domain is highly conserved but the intrinsically disordered C-terminal tail is divergent. **a** *E. coli* photolyase and the PHR domains of *Arabidopsis* CRY1, *Drosophila* CRY, and human CRY1 (solid rectangle) are highly conserved and share an α/β domain containing a secondary pocket (yellow) that can non-covalently bind a secondary antenna chromophore such as MTHF, and an α-helical domain that contains an FAD-binding pocket (green). Not all CRYs bind both chromophores, and while *Drosophila* CRY binds FAD, human CRY1 does not co-purify with chromophores. Photolyase lacks a C-terminal tail, while cryptochromes from diverse species possess divergent tails (dashed rectangles). **b** An overlay of the crystal structures of *E. coli* photolyase (PDB 1DNP, gray) and *Drosophila* CRY (PDB 4 K03, cyan, tail in magenta) highlighting structural similarities. Both possess an FAD-binding pocket that binds FAD (yellow), and a secondary pocket. The secondary antenna chromophore (MTHF, green) binds to the secondary pocket of photolyase but not *Drosophila* CRY. The C-terminal tail of *Drosophila* CRY (magenta) interacts with the FAD pocket. **c** Cartoon demonstrating how the PHR/tail (cyan/magenta) interaction yields a reversible autoinhibited state. CRY is in an active state when the PHR domain and tail are unbound from each other

Across the kingdoms of life, the photolyase/cryptochrome family helps organisms respond or adapt to environmental stresses set in place as the sun rises and sets each day. DNA photolyases utilize a flavin adenine dinucleotide (FAD) co-factor to harvest blue light and catalyze the repair of UV-induced DNA lesions such as cyclobutane pyrimidine dimers or pyrimidine-pyrimidone (6–4) photoproducts [1, 2]. In contrast to their more ancient photolyase homologs, cryptochrome (CRY) proteins are defined by the loss of DNA repair activity, while they have gained other functions in cell signaling [3]. The N-terminal domain of CRY, known as the photolyase homology region (PHR), is structurally similar to photolyase; both are composed of an N-terminal α/β subdomain that forms a secondary pocket that binds an antenna chromophore such as 5,10-methenyl-tetrahydrofolate (MTHF), and a C-terminal helical subdomain that contains the FAD-binding pocket [4–6] (Fig. 1). Some CRY PHRs, such as those from mammals, do not co-purify with chromophores, while other CRY PHRs from plants, insects or vertebrates do [7–11]. Therefore, depending on co-factor binding, chromophore-binding CRYs have photoreceptive functions while others function independently of light.

One way in which all cryptochromes differ structurally from photolyase is through the acquisition of a C-terminal intrinsically disordered region (IDR) known simply as the CRY tail (Fig. 1). In contrast to the highly conserved PHR domain, the tail is highly divergent between CRY paralogs and across different species [3]. Changes to the FAD-binding pocket and the evolution of a C-terminal tail in CRYs may be related to their loss of DNA repair activity. For example, the flexible tail of *Drosophila* CRY (dCRY) uses a hydrophobic motif to dock into its FAD-binding pocket in a manner that is similar to the (6–4) photolesion substrate binding to photolyase [7, 8] (Fig. 1b). In the absence of light, dCRY is maintained in this docked and autoinhibited state, which prevents complex formation of the PHR domain with its clock protein target TIMELESS (dTIM) [12–14] and the E3 ubiquitin ligase JETLAG [15]. Light induces photoreduction of the FAD co-factor to alleviate the dCRY PHR/tail interaction, allowing the dCRY PHR domain to regulate the proteasomal degradation of dTIM [16]. Since levels of dTIM represent an important state variable of circadian rhythms in *Drosophila*, the dCRY photoreceptor thus entrains circadian rhythms to the environment via light. The photoreceptive function of dCRY is mediated by a transition of the dCRY tail from order to disorder [7], suggesting that the dCRY tail exhibits cryptic disorder and unfolds to allow dCRY to perform its function [17]. There is now evidence that the presence of an intrinsically disordered C-terminal tail and ability to reversibly bind its respective PHR domain are defining features of CRY function from plants to insects to mammals [14, 18, 19] (Fig. 1c).

**Fig. 2:**
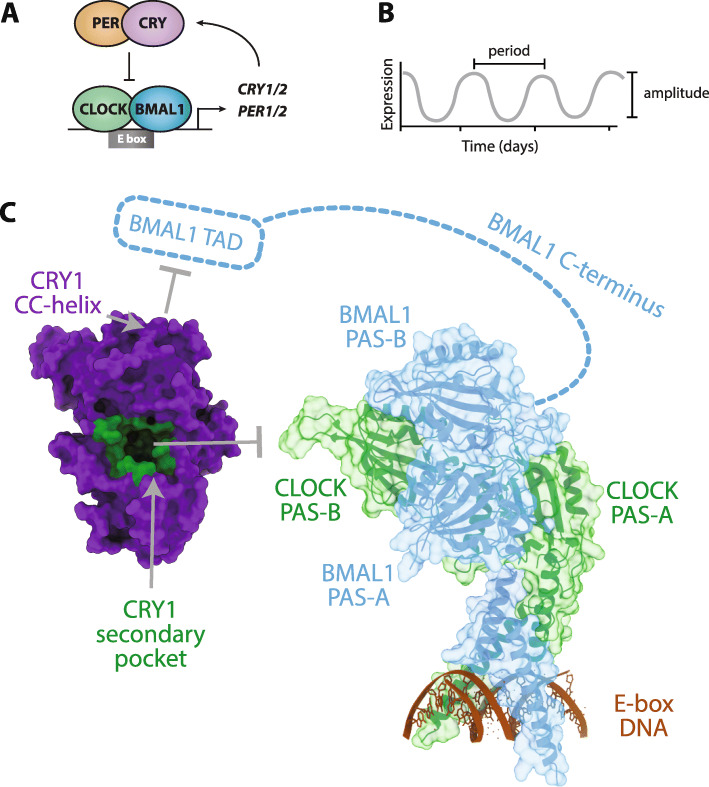
Circadian rhythms are driven by a transcription-translation feedback loop to control rhythmic gene expression. **a** The transcription factor CLOCK:BMAL1 (green/blue) promotes transcription of its repressors PER (light orange) and CRY (purple), which ultimately feedback to bind CLOCK:BMAL1 and repress its activity in the core transcription-translation loop of the mammalian circadian clock. **b** The circadian clock results in rhythmic gene expression on the circadian timescale. The distance between peaks determines the period, while the distance from peak to trough is measured as the amplitude, or the strength of the timekeeping cue. **c** Crystal structure of CRY1 (PDB 5T5X, purple) and CLOCK:BMAL1 (PDB 4F3L, CLOCK in green, BMAL1 in cyan) highlighting regions and domains that interact with each other. The CRY1 CC-helix interacts with the BMAL1 TAD (cyan dashed rectangle) on the disordered BMAL1 C-terminus (cyan dashed line) and the CRY1 secondary pocket (dark green) interacts with CLOCK PAS-B

This commentary aims to discuss recent findings regarding the disordered C-terminal tail in mammalian CRYs and its role in regulating circadian rhythms. In mammals, CRYs serve as light-independent transcriptional repressors in the transcription-translation feedback loop that generates circadian rhythms [20]. The heterodimeric transcription factor CLOCK:BMAL1, composed of circadian locomotor output cycles kaput (CLOCK) and brain and muscle, ARNT-like protein 1 (BMAL1), promotes the transcription of its own repressors, PERIOD (PER1/PER2) and CRY (CRY1/CRY2) [21] (Fig. 2a). This core feedback loop is interlocked with several interdependent transcriptional feedback loops that collectively regulate approximately 40% of the genome in a circadian manner, giving rise to an oscillation of gene expression with a circadian period of about 24 h [22] (Fig. 2b). Because of the widespread nature of circadian transcriptional control, the disruption or alteration of circadian rhythms is linked to metabolic, cardiovascular, psychiatric and sleep-phase disorders [23]. For instance, one prevalent human allele affects splicing of the CRY1 tail (*CRY1Δ11*) to cause a form of familial delayed sleep phase disorder (DSPD) [24]. Although this recent study provides compelling evidence that the CRY1 tail plays a powerful role in regulating human physiology and behavior, relatively little is known about the mechanism(s) by which the tails regulate cryptochrome function in mammals. While crystal structures have laid the foundation for a mechanistic understanding of how the PHR domains of CRY1 and CRY2 generate and regulate circadian rhythms [7, 10, 25–28], none of these structures have shed light on the role of mammalian CRY tails. Recently, a combination of biochemical studies, innovations in protein NMR spectroscopy, and new chemical biology tools have begun to shed some light on CRY tail function in circadian rhythms.

## Main text

### Mapping CRY function from its PHR domain to its unstructured tail

The CRY PHR domain interacts with two distinct sites on the transcription factor CLOCK:BMAL1 to directly repress its activity. During the day, CLOCK:BMAL1 recruits transcriptional co-activators like CREB-binding protein (CBP) or p300 through the transactivation domain (TAD) on the distal C-terminus of BMAL1 [29, 30]. CRY proteins accumulate and enter the nucleus in the evening, allowing the coiled-coil (CC)-helix on the PHR domain to interact directly with the BMAL1 TAD [30, 31] (Fig. 2c). Competition for mutually exclusive binding sites on BMAL1 allows CRY to sequester the TAD from co-activators; however, the affinity of CRY1 for the TAD is modest, so an additional interaction that stabilizes CRY recruitment to the transcription factor is required for effective repression [30]. Recruitment is mediated by the secondary pocket on the PHR domain, which docks onto the PER-ARNT-SIM (PAS) domain core of CLOCK:BMAL1 (composed of CLOCK PAS-A, CLOCK PAS-B, BMAL1 PAS-A BMAL1 PAS-B), specifically through the CLOCK PAS-B domain [25, 26] (Fig. 2c). The multivalent binding of CRY to CLOCK:BMAL1 is important, as mutants that decrease affinity for the BMAL1 TAD or CLOCK PAS-B significantly reduce CRY-mediated repression, while mutation of both interfaces completely eliminates repression [32, 33]. A few amino acid substitutions fine-tune protein dynamics at the secondary pocket interface between the two mammalian CRY paralogs to contribute to their differential affinity for the PAS domain core [25] and largely confers paralog-specific effects on circadian period [34]. Altogether, quantitative analyses of CRY, CLOCK and BMAL1 mutants support an emerging model in which circadian period is correlated with the affinity of CRY for CLOCK:BMAL1––the higher the affinity that CRY has for the CLOCK:BMAL1, the longer circadian period becomes [30].

**Fig. 3:**
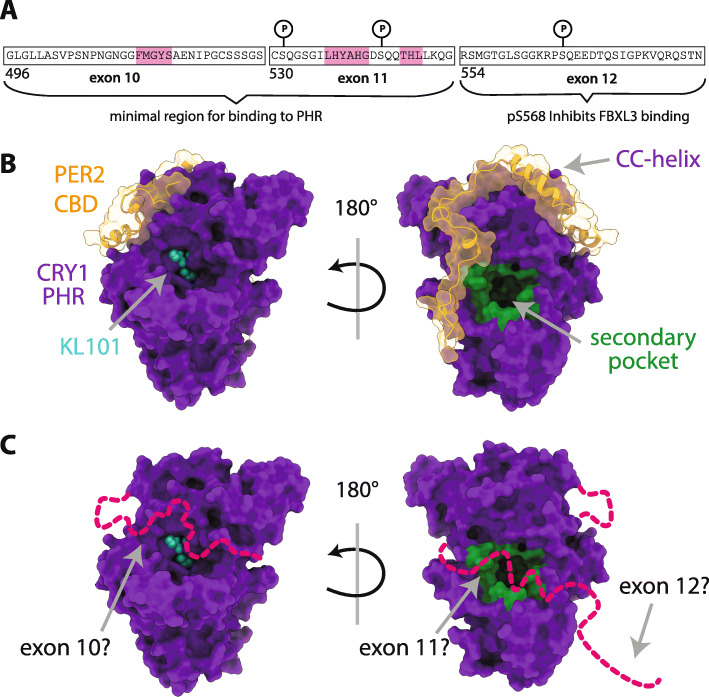
The human CRY1 tail regulates multiple sites on the PHR domain. **a** The hCRY1 tail is composed of exons 10 (residues 496–529), exon 11 (residues 530–553), and exon 12 (residues 554–586). NMR chemical shift mapping identified distinct linear motifs in exons 10 and 11 (pink) that may be involved in binding directly to the PHR domain [35]. DNA-PK-dependent phosphorylation sites in exons 11 and 12 are marked below a circled ‘P’ [36]. **b** The PER2 CBD (orange) wraps around the CRY1 PHR domain (purple) near regions where the hCRY1 tail might also interact, such as the CC-helix, FAD-binding pocket (KL101 is a CRY1-selective ligand, cyan) and the secondary pocket (green) in PDBs 6KX6, KL101-bound and 6OF7, PER2 CBD-bound. Thin dashed lines indicate flexible regions on the PHR domain that were missing density. **c** A possible model for how the tail (magenta) might bind to both the FAD-binding pocket and the secondary pocket on the PHR domain in the absence of PER proteins. Exon 10 has been implicated at binding near the FAD-binding pocket [37], while exon 11 inhibits CLOCK PAS-B binding at the secondary pocket and deletion of exon 12 has no effect on affinity of the tail for the PHR domain [35]

Even though the CRY PHR domain makes critical contacts with CLOCK:BMAL1 to directly repress its activity, numerous studies over the last decade have demonstrated that the CRY C-terminal tails also contribute to regulation of circadian rhythms. In human CRY1 (hCRY1), the intrinsically disordered C-terminal tail is 95 amino acid residues long, including part of exon 10 and the entire length of exons 11 and 12, while the mouse CRY1 (mCRY1) tail is a bit longer due to a 20 amino acid duplication within exon 10 (Fig. 3a). The CRY2 tail is a bit shorter at only 80 residues in length and highly divergent in sequence from the CRY1 tail [3]. While genetic complementation studies have established that the CRY PHR domain is necessary and sufficient to reconstitute circadian rhythms in *Cry1*^*−/−*^*;Cry2*^*−/−*^ cells, the absence of C-terminal tails affects the amplitude and period of reconstituted rhythms [38, 39]. Furthermore, swapping the CRY2 tail onto the CRY1 PHR domain is sufficient to shorten circadian period, while swapping the CRY1 tail onto CRY2 lengthens circadian period in these rescue assays [38], suggesting that sequences encoded by one or both of these tails have the capability to modulate the function of the PHR domains, possibly in different ways.

Post-translational modifications of both CRY tails also influence circadian rhythms, providing the first clues as to how the function of these intrinsically disordered tails could be regulated in vivo. IDPs and IDRs are typically enriched for post-translational modifications and this is most likely due to their accessibility as substrates for modifying enzymes or as binding partners for cell signaling [40]. Priming phosphorylation of the CRY2 tail at S557 by the dual specificity tyrosine-phosphorylation-regulated kinase 1A (DYRK1A) and subsequent phosphorylation upstream at S553 by glycogen synthase kinase 3β (GSK-3β) promotes the proteasomal degradation of CRY2 [41, 42]. Ablating this phosphorylation with the CRY2 S557A mutant lengthens circadian rhythms in vivo, most likely due to stabilization of CRY2 [43]. Similarly, phosphorylation at mouse CRY1 at S588 or its corresponding phosphomimetic mutation, S588D, have a stabilizing effect against proteasomal degradation and extend circadian period [36]. Stabilization of the CRY1 S588D mutant against proteasomal degradation is likely mediated by decreased association with FBXL3 [44, 45], a substrate adaptor for Skp-Cullin-Fbox (SCF) E3 ligases, and a concomitant increase in association with the deubiquitinase USP7 (also known as the Herpesvirus-associated ubiquitin-specific protease, HAUSP) [45]. In addition, phosphorylation of S588 affects the intracellular distribution and rate of nuclear entry of CRY1 [39, 46], which may result from the proximity of S588 to a potential nuclear localization sequence (NLS) on the tail [47]. Mutations at this putative NLS, such as the K585A/R586A double mutant, affect cellular distribution of mCRY1 [47]; however, it has yet to be determined whether the K585A/R586A mutant influences circadian rhythms. Therefore, the intrinsically disordered tails are regulated reversibly by phosphorylation to influence the stability and intracellular distribution of cryptochromes.

### Night owls provide the first clues how the CRY1 tail controls human circadian timing

Recently, the intrinsically disordered tail of CRY1 was linked directly to regulation of circadian timekeeping and delayed sleep phase disorder in humans by discovery of a mutant allele in the *CRY1* gene [24]. Delayed sleep phase disorder is characterized by later than normal sleep onset times (a.k.a. night owl behavior), which is often linked to a longer than average circadian period [48]. The mutant allele is caused by a single nucleotide polymorphism, *CRY1* c.1657 + 3A > C, also known as *CRY1Δ11*, that alters the 5′ splice site downstream from exon 11 and leads to alternate splicing that removes the 24 amino acids encoded by exon 11 from the disordered tail [24] (Fig. 3a). The delayed sleep phase disorder phenotype is autosomal dominant and manifests in carriers of the mutant allele, which occurs with a frequency of 0.1 to 0.6% in the human population, and as high as 1 in 75 people in certain populations [24]. The discovery of this mutant allele, with its powerful control over circadian rhythms and sleep timing, offered an unprecedented opportunity to explore the mechanism by which the intrinsically disordered tail regulates CRY1 function.

People with the *CRY1Δ11* allele have an increase in the period of their circadian rhythms, which is recapitulated in cellular studies by genetic complementation of the mutant allele in *Cry1*^*−/−*^*;Cry2*^*−/−*^ mouse fibroblasts [24]. Strikingly, CRY1Δ11 is a more effective transcriptional repressor of CLOCK:BMAL1 than wild-type CRY1, as determined by chromatin occupancy and expression profiles of CLOCK:BMAL1-dependent genes [24]. Enhanced repression by the mutant allele could arise from increased stability of the mutant protein, enhanced nuclear entry or retention, and/or tighter binding to CLOCK:BMAL1. Wild-type and mutant proteins exhibited similar stability in cellular assays, but CRY1Δ11 had a moderate increase in nuclear accumulation compared to wild-type protein [24]. In addition, CRY1Δ11 appeared to bind CLOCK:BMAL1 more tightly, as determined by enhanced association with CLOCK:BMAL1 in co-immunoprecipitation assays [24]. These findings are consistent with the emerging model that tighter binding of CRY leads to a longer circadian period [30]. Because the mutant allele directly modifies only the intrinsically disordered C-terminal tail, and not the PHR domain where cryptochromes have been shown to directly interact with CLOCK:BMAL1, these data suggest that loss of exon 11 relieves some type of autoinhibitory role of the tail between the CRY1 PHR domain and CLOCK:BMAL1, thus making CRY1Δ11 a more effective repressor.

### The CRY1 tail serves an autoinhibitory function that controls affinity for CLOCK:BMAL1

Over the last two decades, numerous groups have demonstrated that PHR/tail interactions serve autoinhibitory roles in cryptochromes from other species, such as *Drosophila* [12–14] and *Arabidopsis* [19]. Experimental evidence for a direct interaction between the PHR domain of a mammalian CRY and its disordered tail was first published in 2005 [18]. A recent study revisited intramolecular interactions of mammalian cryptochromes in more depth with fluorescence polarization (FP) binding assays and nuclear magnetic resonance (NMR) spectroscopy to identify the molecular determinants by which the CRY1 tail directly interacts with its PHR domain [35].

It can be quite challenging to study long IDPs at atomic resolution with conventional protein NMR methods such as the ^1^H-^15^N heteronuclear single quantum coherence (HSQC) experiment due to the lack of unique chemical environments in the disordered protein that severely restrict peak dispersion in the ^1^H dimension of HSQC spectra [18, 35]. By contrast, innovative direct ^13^C-detected experiments such as the ^13^C-^15^N CON not only increase peak dispersion, but also allow for detection of proline residues that are typically enriched in IDPs, thus improving biophysical studies of long, native-like IDPs [49]. Related ^13^C-detected triple resonance experiments such as the 3D (HACA) N (CA) NCO and (HACA) N (CA) CON aid in assigning peaks for individual residues for long IDPs [49]. The use of CON NMR methods was critical for enabling the identification of residues on the CRY1 tail that exhibit chemical shift perturbations upon addition of the PHR domain; these residues are primarily centered in short linear motifs in exons 10 and 11, which also correspond to the minimal regions on the tail needed for binding to the PHR domain by FP-based binding assays [35] (Fig. 3a). This highlights the powerful role that NMR spectroscopy can play in the study of intrinsically disordered proteins by providing atomic resolution data to map binding and quantify the dynamics of IDPs in the absence of crystal structures.

Although NMR mapping studies identified PHR-dependent chemical shift perturbations in exons 10 and 11 of the intact CRY1 tail, exon 11 is necessary and sufficient to complete with CLOCK:BMAL1 binding in vitro [35]. Given that CLOCK:BMAL1 engages with two distinct binding sites on the CRY1 PHR domain [25, 26, 30], both interactions were explored as potential site(s) of competition; exon 11 clearly competes with the CLOCK PAS-B for the CRY1 secondary pocket, and not the interaction between the CRY1 CC-helix and the BMAL1 TAD [35]. Interestingly, exon 11 shares properties with other intrinsically disordered inhibitory modules, such as a similar length (10–40 amino acids) and a susceptibility to being altered in splicing events [50]. The mutant allele gives rise to CRY1Δ11 protein, which lacks this autoinhibitory module and binds the transcription factor several-fold tighter [35], thus providing a molecular rationale for increased repression of CLOCK:BMAL1 and *CRY1Δ11*-mediated delayed sleep phase disorder [24].

Compared to both CRY1Δ11 and the isolated PHR domain, full-length CRY1 has a decreased association rate constant (*k*_on_) rather than an increased dissociation rate constant (*k*_off_) for binding to the PAS domain core of CLOCK:BMAL1 [35]. This decrease in *k*_on_ rather than an increase in *k*_off_ is seen other proteins that contain an autoinhibitory domain tethered by a flexible linker [51]. Moreover, the decreased *k*_on_ for CRY1 falls within the diffusion-limited range (10^4^–10^6^ M^− 1^ s^− 1^) [52], suggesting that the flexible tail interacts transiently with the PHR domain to regulate association with CLOCK:BMAL1. However, it is still not known exactly where on the PHR domain or exon 11 that the two make direct contact. It is possible that the CRY1 tail regulates CLOCK:BMAL1 binding through an allosteric mechanism, although it seems parsimonious to suggest that exon 11 and CLOCK PAS-B have overlapping binding sites in or near the CRY1 secondary pocket.

### Regulation of CRY1 by its disordered tail is influenced by other clock protein factors

Other clock protein components, such as the co-repressor PERIOD (PER), also associate with CRY PHR domains to modulate their association with CLOCK:BMAL1 [25, 34]. The approximately one-hundred residue-long CRY-binding domain (CBD) of PER2 is also intrinsically disordered, but forms several short helices upon association with CRY1 or CRY2, where it wraps in an extended fashion around the PHR domain in proximity to both the CC-helix and the secondary pocket where CLOCK PAS-B (and possibly CRY1 exon 11) binds [25, 27, 28] (Fig. 3b). The PER2 CBD efficiently displaces the isolated CRY1 tail from binding to its PHR domain, suggesting that the PER2 CBD and the CRY1 tail have at least some overlap in their binding sites on the PHR domain [35] (Fig. 3c). While both are intrinsically disordered, PER2 CBD binds to CRY1 with nanomolar affinity [28], while the CRY1 tail binds to its PHR domain with low micromolar affinity in *trans* [35]. While the intrinsically disordered PER2 CBD becomes partially folded when bound to CRY1 [25, 27, 28], it is not known whether the CRY1 tail exhibits similar conformational changes. If the CRY1 tail undergoes a transition to a partially folded or ordered state when bound to the CRY1 PHR, then competitive interactions with a PER binding partner could awaken the cryptic disorder of the CRY1 tail [17]. The striking ability of PER2 to compete with the tail for the PHR domain also suggests a specific window of temporal regulation within the 24-h day for regulation of CRY1 by its intrinsically disordered tail. CRY1 is associated with PER proteins, CRY2, and other epigenetic factors in the ~ 1.9 MDa ‘early’ repressive complex [53], whereas CRY1 represses CLOCK:BMAL1 without PER proteins in the ‘late’ repressive complex at the initiation of each circadian cycle at dawn [54]. Therefore, differences in activity between CRY1 and CRY1Δ11 with regards to the autoinhibitory tail are likely most pertinent in the context of the ‘late’ repressive complex when PER proteins are absent.

The role for post-transcriptional and post-translational modifications in modulating the CRY1 PHR/tail interaction needs to be explored further. Several DNA-PK-dependent phosphorylation sites have been identified in the CRY1 tail [36] (Fig. 3a). Of these, two sites flank exon 11 and the third is located at residue S588 (mouse numbering; residue 568 in hCRY1). The role of the two phosphorylation sites near exon 11 has not yet been studied, but the phosphomimetic mutation S588D that stabilizes CRY1 protein, located in exon 12 of the tail, does not affect the affinity between the CRY1 tail and the PHR domain [35]. This suggests that any phenotypic effects of the CRY1 S588D mutant in extending CRY1 half-life and circadian period might be independent of the PHR/tail interaction. On the other hand, the S588D mutation could subtly affect the conformation of the CRY1 tail to antagonize access of the ubiquitin ligase FBXL3 to the FAD-binding pocket of the PHR domain thus protecting CRY1 from proteasomal degradation [44, 45]. Finally, there is also a possibility that alternative splicing could regulate CRY1 tail function be deleting exon 11 in a signal or tissue-specific manner. Other IDRs involved in autoinhibition are often regulated by alternate splicing events to modulate protein interactions and cell signaling pathways [50, 55]. It remains to be seen whether regulated alternative splicing in a wild-type *Cry1* genetic background could yield CRY1Δ11 protein to modulate circadian timing. The human CRY1Δ11 allele is therefore an example of a permanently altered splicing event in which exon 11 is skipped, thus removing an autoinhibitory module from the tail, and altering the circadian period [24].

### The circadian clock can be modulated by small molecules that rely on CRY tails

Nearly a decade ago, cryptochromes entered the chemical biology arena with the discovery of KL001, a small molecule that stabilizes CRY half-life by occupying the FAD-binding pocket of CRY to compete with binding of the ubiquitin ligase FBXL3 [10, 56, 57]. More recently, Hirota and colleagues identified two new molecules that target CRY1 or CRY2 to extend circadian period by selectively extending the half-life of their respective target [37]. Although they similarly bind to the highly conserved FAD-binding pocket of their respective CRY targets, both small molecules require the intrinsically disordered tails for selectivity and the stabilization of cryptochromes in cells [37]. To identify how the tails contribute to selectivity, chimeras swapping regions of the tail between CRY1 and CRY2 revealed that the PHR domain-proximal exon 10 is required for selectivity in cells [37]. Given that the molecules target the FAD-binding pocket on the opposite side of the PHR domain from the secondary pocket, these data suggest that the short, linear binding motifs identified in exons 10 and 11 may target discrete sites on the PHR domain (Fig. 3c). Overall, the intrinsically disordered tails of cryptochromes now appear to be compelling new targets for modulating the circadian clock.

## Conclusions

As direct repressors of CLOCK:BMAL1 transcriptional activity, cryptochromes sit at the nexus of circadian rhythms as central regulators of the vast transcriptional network of the mammalian circadian clock [58]. With these new insights into cryptochrome structure and dynamics, we are now poised to understand the roles that their intrinsically disordered tails play in fine-tuning circadian timing. Similar to cryptochromes from other species [9, 12, 13, 19], the mammalian CRY1 tail also binds to its PHR domain to form an autoinhibited conformation [35]. Identifying the autoinhibitory function of exon 11 and a molecular rationale for *CRY1Δ11*-mediated delayed sleep phase disorder should motivate the development of inhibitors that modulate the CRY1/CLOCK interaction at the secondary pocket to restore normal circadian timing (and sleep onset) or reset circadian rhythms [24, 35]. Conversely, small molecule ligands that target the FAD-binding pocket to modulate circadian rhythms also appear to suggest an important functional role for CRY tails near the FAD-binding pocket [37].

New insights into the critical role of the CRY1 tail should pave the way for understanding how the divergent intrinsically disordered tails contribute to functional differences between CRY1 and CRY2. For instance, while the respective PHR domains of CRY1 and CRY2 share 80% identity, their tails are highly divergent. Swapping as few as seven residues between the CRY1 and CRY2 PHR domains can make one paralog resemble the other with regards to CLOCK:BMAL1 binding [25, 34]. However, to completely recapitulate paralog-specific effects on circadian period, the tails of each paralog also need to be swapped [34], highlighting the remarkable role that these intrinsically disordered regions play in regulation of circadian timing and beyond.

For instance, post-translational modifications of the CRY1 tail help shift the phase of circadian rhythms after DNA damage; exposure to genotoxic stress increases phosphorylation of the mouse CRY1 tail at S588, thus stabilizing CRY1 to extend circadian period length [45]. Studying this and other factors that modify or compete for the CRY tails will provide further insights into how intrinsically disordered CRY tails act as signaling hubs to transduce signals between the molecular circadian clock and different pathways.

## Abbreviations

BMAL1: Brain and muscle ARNT-like protein 1
CBD: Cryptochrome binding domain
CBP: CREB-binding protein
CC-helix: Coiled-coil helix
CLOCK: Circadian locomotor output cycles kaput
CRY: Cryptochrome
CRY1: Cryptochrome 1
CRY2: Cryptochrome 2
CT: Circadian time
dCRY: *Drosophila* CRY
DYRK1A: Dual specificity tyrosine-phosphorylation-regulated kinase 1A
DNA: Deoxyribonucleic acid
DNA-PK: DNA-dependent protein kinase
DSPD: Delayed sleep phase disorder
dTIM: Drosophila TIMELESS
FAD: Flavin adenine dinucleotide
FBXL3: F-box/LRR-repeat protein 3
FP: Fluorescence polarization
GSK-3β: Glycogen synthase kinase 3β
HAUSP: Herpesvirus-associated ubiquitin-specific protease
hCRY1: Human cryptochrome 1
HSQC: Heteronuclear single quantum coherence
IDP: Intrinsically disordered protein
IDR: Intrinsically disordered region
KIX: Kinase-inducible domain (KID) interacting (domain)
*k*_off_: Dissociation rate constant
*k*_on_: Association rate constant
mCRY1: Mouse cryptochrome 1
MTHF: 5,10-methenyl-tetrahydrofolate
NMR: Nuclear magnetic spectroscopy
PAS: PER-ARNT-SIM (domain)
PAS-B: PAS domain B
PER: Period (protein)
PER1: Period circadian protein homolog 1
PER2: Period circadian protein homolog 2
PHR: Photolyase homology region
SCF: Skp-Cullin-Fbox
TAD: Transactivation domain
